# Wogonin modulates hydroperoxide-induced apoptosis via PI3K/Akt pathway in retinal pigment epithelium cells

**DOI:** 10.1186/s13000-014-0154-3

**Published:** 2014-11-29

**Authors:** Tingqin Yan, Hongsheng Bi, Yun Wang

**Affiliations:** Clinical College of Shandong University of Traditional Chinese Medicine, Jinan, 250355 China; Central Hospital, Shandong Province, Taian, Shandong 271000 China; Eye Hospital of Shandong University of Traditional Chinese Medicine, Jinan, 250002 China

**Keywords:** Age-related macular degeneration, Hydrogen peroxide, Oxidative stress, Retinal pigment epithelial cell, ARPE-19

## Abstract

**Background:**

Oxidative stress causes the defects of retinal pigment epithelial (RPE) cells that contribute to age-related macular degeneration (AMD). This study was conducted to determine whether wogonin could prevent H_2_O_2_-induced oxidative stress in RPE cells.

**Methods:**

A RPE cell line, ARPE-19, was obtained for the cell model. ARPE-19 cells were pre-treated with various concentrations of wogonin for 24 h before being exposed to H_2_O_2_ for 2 h to induce oxidative stress. Cell metabolic activity was measured using 3-(4,5-dimethylthiazol-2-yl)-2,5-diphenyltetrazolium bromide (MTT) assay. Cellular apoptosis was quantified by the flow cytometry. Protein level was assed by western blot.

**Results:**

The RPE cells exposed to to 200 mM H_2_O_2_ demonstrated a significant depression in the cell viability; whereas pre-treatment with 50 and 100 mmol/l wogonin could significantly improve the cell viability in a dose-dependent manner. The proportion of PI-positive cells was increased significantly in RPE cells treated with H_2_O_2_ alone; whereas pretreatment with 100 mM wogonin significantly reduced H_2_O_2_ -induced RPE cell death rate. In protein level, the wogonin use could reduce the level of p-Akt significantly and this is the possible mechanism of the antioxidant effect of wogonin.

**Conclusions:**

Our study showed that wogonin pre-treatment can protect RPE cells from H_2_O_2_-induced apoptosis. This suggests potential effect of wogonin in the prevention of retinal diseases associated with H_2_O_2_-induced oxidative stress such as AMD.

**Virtual Slides:**

The virtual slide(s) for this article can be found here: http://www.diagnosticpathology.diagnomx.eu/vs/13000_2014_154

## Background

Age-related macular degeneration (AMD) is the leading cause of visual loss among the elderly in the developed countries [1,2]. Oxidative stress is considered to play an important role in the retinal pigment epithelial (RPE) death during aging of RPE and the pathogenesis of AMD [3]. RPE layer is a single cellular layer that forms the outer blood-retinal barrier [4]. It is essential as a nutritional or metabolic support for photoreceptor and phagocytosis. RPE is particularly susceptible to oxidative stress by reactive oxygen species (ROS), such as superoxide anion, hydroxyl radical, singlet oxygen, and H_2_O_2_, due to its locations and functions. Dysfunction of RPE contributes to retinal diseases such as retinitis pigmentosa (RP) and AMD leading to visual impairment [5,6]. H_2_O_2_ is produced during RPE phagocytosis of photoreceptor outer segments and is generated as a consequence of light stimulation [7,8]. H_2_O_2_ is also generated by the photo excited pigment lipofuscin which accumulates during aging in the RPE cells and the age-related accumulation of lipofuscin within the RPE is strongly associated with the development of AMD [9]. The catalase activity of RPE, which neutralizes H_2_O_2_, is significantly reduced in eyes with AMD [10]. A number of *in vitro* studies have shown that oxidative stress induced by H_2_O_2_ would lead to RPE damage, with preferential damages to mitochondrial DNA and RPE cell death. Evidence for the involvement of oxidative stress in RPE death during aging and AMD comes also from the observation that daily administration of antioxidant nutrients may reduce the risk of AMD, which may result in significant health-care cost savings [11,12]. Antioxidants that may play an important role include flavonoids, which are polyphenolic compounds present in high concentrations in certain plants, fruits, vegetables and other plant-derived foods [13].

**Figure 1:**
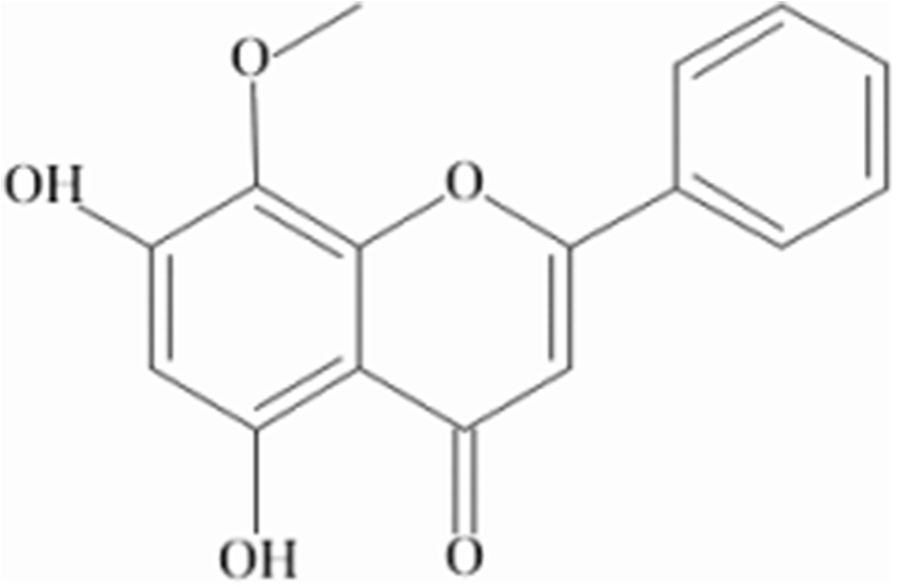
**The chemical structural formula of wogonin.**

Flavonoids, a class of plant secondary metabolites, are the most common group of polyphenolic compounds that are ubiquitous in vegetables, fruits, and some medicinal plants. Flavonoids are recognized as anti-oxidant or anti-tumor agents. Wogonin is a naturally flavonoid isolated from the root of *Scutellaria baicalensis Georgi*, which has been widely used for its antioxidant, anti-inflammatory, and anticancer activities (Figure 1) [14]. The anticancer activity of wogonin has been reported various human cell lines including myeloma cell RPMI 8226, hepatocellular carcinoma SK-HEP-1 and SMMC-7721, human breast cancer cells and human cervical carcinoma HeLa cells [15]. Its activity is mediated by the cancer cell differentiation, and is regulated by various genes and proteins [16]. However, the effect of wogonin on the H_2_O_2_ induced RPE cells is still unclear. We therefore evaluated the effect of wogonin on cellular events associated with AMD on RPE cells in vision research in an *in-vitro* model.

## Methods

Cell Titer 96 One Solution Cell Proliferation Assay (MTT) reagent was purchased from Promega (Madison, WI, USA). Phenazine methosulfate (PMS), Hoechst stain solution, and propidium iodide (PI) were obtained from Sigma (St. Louis, MO). NE-PER® nuclear and cytoplasmic extraction reagents were purchased from Thermo Scientific (Rockford, IL). A fluorescein isothiocyanate (FITC) annexin V Apoptosis Detection Kit I and phospho-pRb antibodies were purchased from BD Biosciences (San Diego, CA). Anti-rabbit human phosphorylation protein kinase B (*p*-AKT) monoclonal antibody and anti-rabbit human β-actin monoclonal antibody are all from American Cell Signaling.

### Cell culture and treatments

ARPE-19 cells, a human RPE cell line, were obtained from American Type Culture Collection (Manassas, VA). The cells were maintained as previously described [17]. The cells were treated with H_2_O_2_ and wogonin for the indicated time periods. After pretreatment with wogonin (Sigma, St Louis, USA, purity ≥ 95%) at concentrations of 50 and 100 mg/l for 24 h, 200 μmol/l H_2_O_2_ was added and incubated with the cells for an additional 24 h. The cells in control group were treated with the same volume of phosphate-buffered saline (PBS), while the cells in model groups were incubated with H_2_O_2_ at 200 μmol/l for 3–24 h. The morphological changes of cells were observed using a phase contrast microscope.

### Cell viability measurement by MTT assay

ARPE-19 cells (1 × 10^5^ cells/well) were seeded and grown in 96-well plates for 24 h. At the designated times, cell viability was determined using MTT assay. Briefly, 100 μl of MTT solution (5 mg/ml) was added to each well and incubated for 4 h at 37°C in the dark. After incubation, 100 μl of dimethylsulfoxide (DMSO) was added to each well to lyse the cells. The absorbance of the sample was measured at 490 nm on a Versamax microplate reader using SoftMax Pro 4.8 analysis Molecular Devices software (Sunnyvale, CA).

### Detection of cell apoptosis by flow cytometry

After the cells are treated, we collect all anchorage-dependent cells and floating cells to the flow cytometer for centrifugation at 1000 RPM for 5 min. Then the cells are washed by PBS twice and record the amount. Then we use 1 × Binding Buffer to re-suspend the cells, adjust the cell concentration to 1 × 10^6^/ml, drip 100 μl of cell suspension to a 5 ml flow tube, and then add Annexin V 5 μl and PI 10 μl to gently blend it and keep in a dark place for 10 min at room temperature. Immediately detect and analyze the result to calculate the percentage of the viable apoptotic cell.

### Western blot assay

Proteins were extracted from RPE cells and the immunblots were probed with primary antibodies for phosphorylated Akt (Ser-473) overnight at 4°C followed by incubation with the corresponding secondary antibodies at room temperature for 1 hour. The blots were visualized with ECL-plus reagent. Phosphorylated Akt immunoblots were then stripped with strip buffer at 50°C for 30 min and re-blotted for total Akt.

### Statistical analysis

The measured data are presented as mean ± standard deviation (x ± sd). We used the t-test to compare the means of two samples and analyzed the dose–response relationship using Spearman rank correlation; the variance analysis was conducted using the statistical software SPSS 13.0. All the results indicating p < 0.05 show statistical significance.

## Result

### Effect of H_2_O_2_ and wogonin on ARPE-19 cell viability

**Figure 2:**
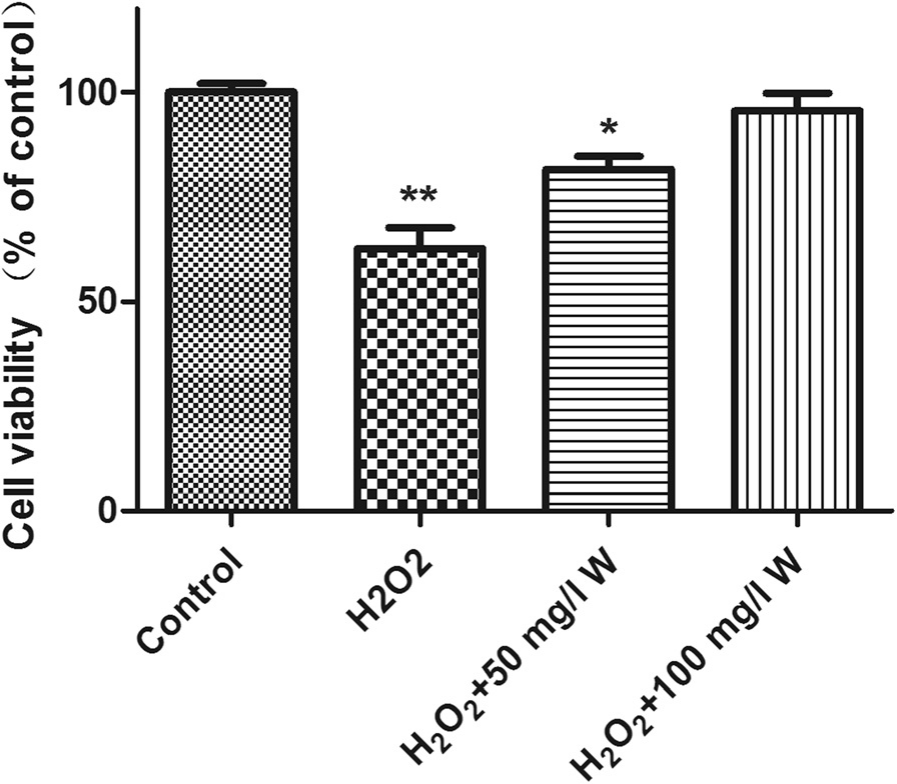
**Effects of wogonin on H**_**2**_ **O**_**2**_ **processed ARPE-19 cells. ARPE-19 cells were preincubated with H**_**2**_ **O**_**2**_ **with or without wogonin.** The cell viability was detected by MTT method. Mean ± SD., n = 6, *: P < 0.05, **P < 0.01 vs. control group.

The viability of ARPE-19 cells treated in control, 200 μmol/l H_2_O_2_, 200 μmol/l H_2_O_2_+ 50 m/l wogonin and 200 μmol/l H_2_O_2_+ 100 m/l wogonin at 24 h were 100% ± 3.0%, 62.7 ± 8.0%, 85.2 ± 3.8% and 95.5 ± 4.4% of the control value, respectively (Figure 2). The viability of ARPE-19 cells treated with 200 μmol/l H_2_O_2_ and 200 μmol/l H_2_O_2_+ 50 m/l wogonin are significantly reduced. However, the 200 μmol/l H_2_O_2_+ 100 m/l wogonin demonstrated non-significant results compared with the control group (Figure 2).

### Wogonin protect APRPE-19 from H_2_O_2_-induced apoptosis

**Figure 3:**
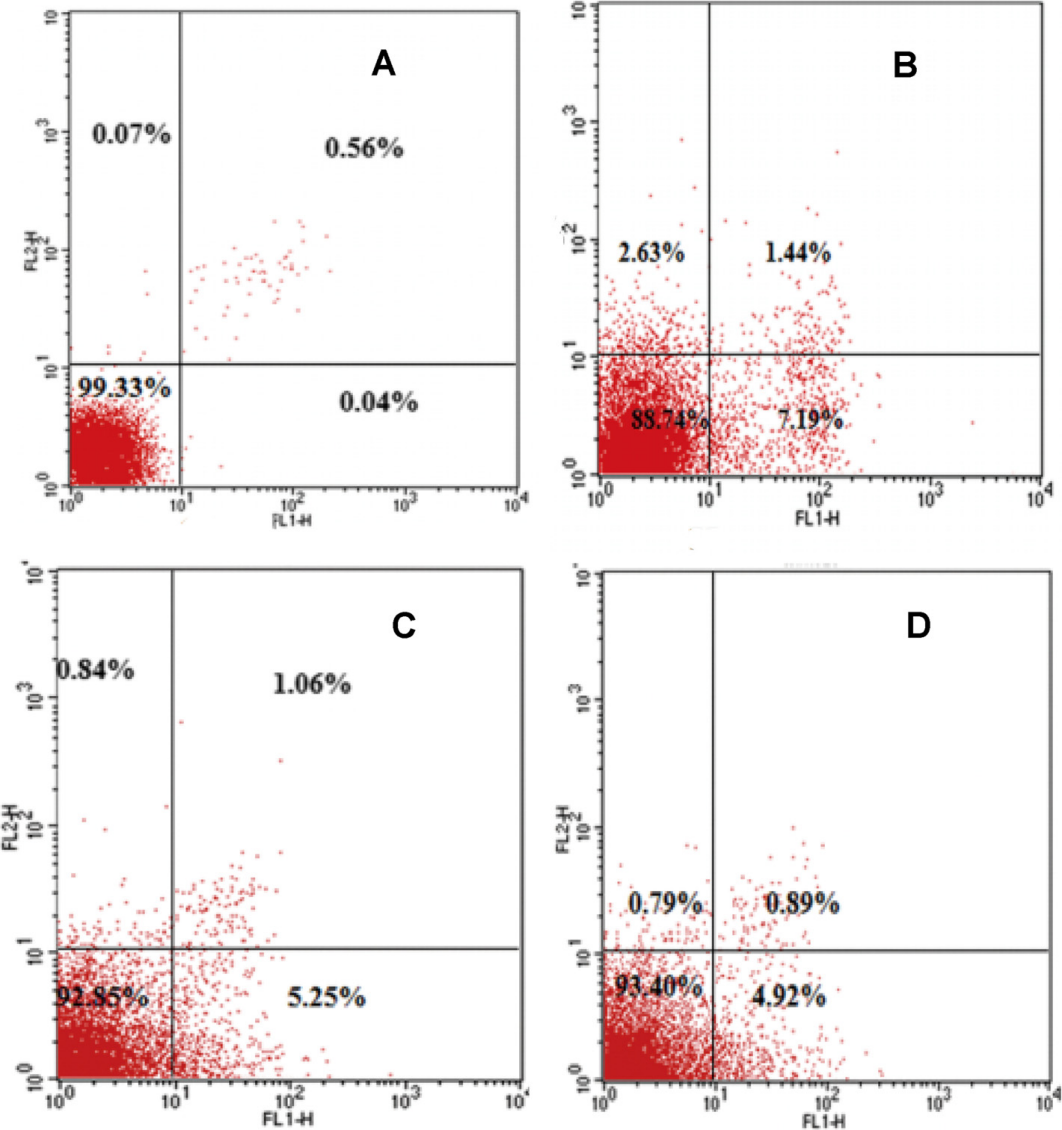
**Modulation in H**_**2**_ **O**_**2**_ **induced apoptosis by wogonin. A**: control group. **B**: 200 μmol/l H_2_O_2._ **C**. 200 μmol/l H_2_O_2_+ 50 m/l wogonin. **D**. 200 μmol/l H_2_O_2_+ 100 m/l wogonin. The S.D. was below 65% in all cases. Results are representative of three separate experiments.

Annexin V-FITC apoptosis detection kit was used to assess the apoptosis effect of wogonin on the H_2_O_2_ induced apoptosis in ARPE-19 cells. As shown in Figure 3A and Figure 3B, compared with the control group (apoptotic rate: 0.04%), 200 μmol/l H_2_O_2_, for 24 h would lead to a significant higher rate of apoptosis. The supplementation of wogonin could reduce the H_2_O_2_ induced apoptosis and a high concentration of wogonin would lead to a more significant effect (apoptotic rate: 4.92%) compared with the lower concentration (apoptotic rate: 5.25%).

### Wogonin down-regulates p-Akt but not total Akt at protein levels

**Figure 4:**
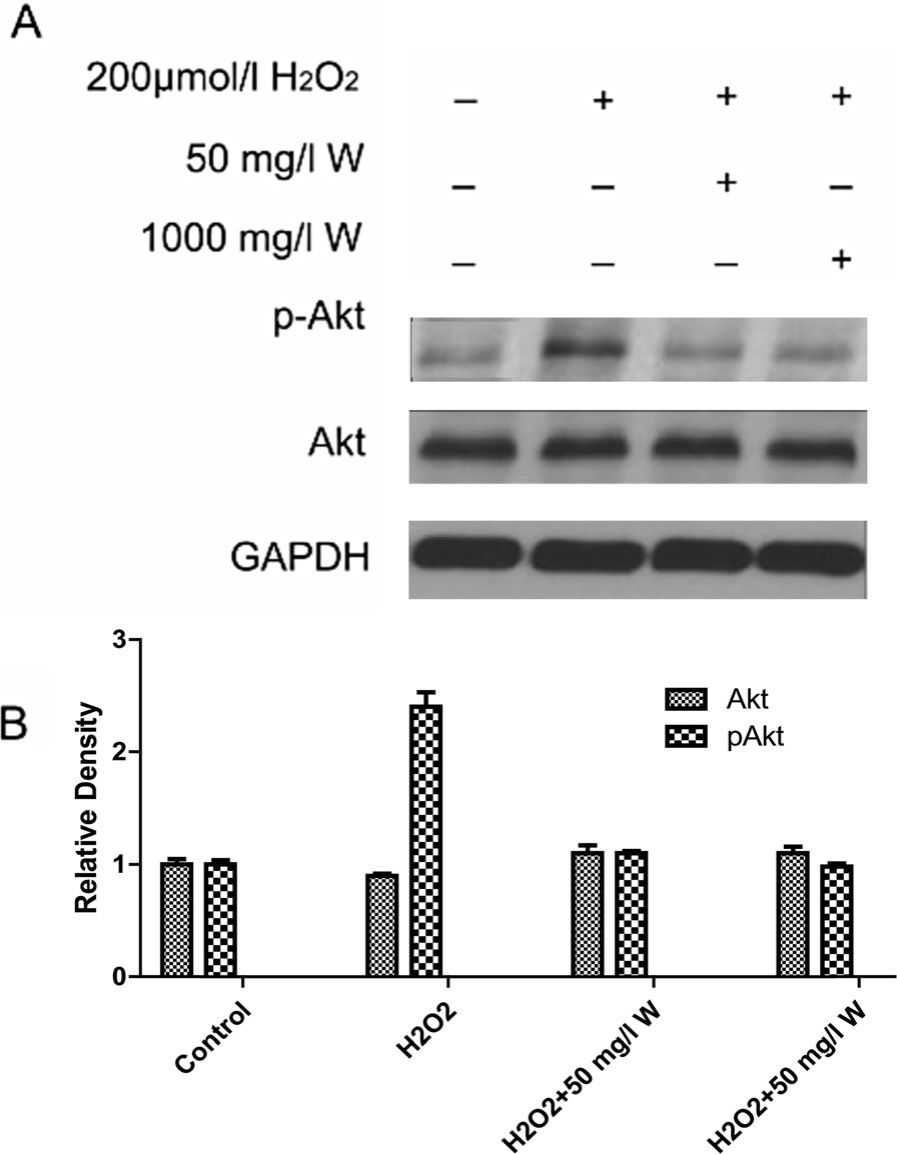
**Phosphorylation and total Akt in ARPE-19 exposed with or without H**_**2**_ **O**_**2**_ **and wogonin. A**. Results are representative of three separate experiments. **B**: Data obtained from quantitative densitometry were presented as mean ± SD of 3 independent experiments. *: P < 0.05, **P < 0.01 vs. control group.

Protein levels of p-Akt were up-regulated in a dose-dependent manner after exposured to different concentrations of wogonin for 48 h (Figure 4a). The relative density of p-Akt in control, 200 μmol/l H_2_O_2_, 200 μmol/l H_2_O_2_+ 50 m/l wogonin and 200 μmol/l H_2_O_2_+ 100 m/l wogonin were 100% ± 0.4%, 62.1% ± 3.0%, 85.2% ± 3.3% and 96.1% ± 3.7%. However, the protein level of Akt was not significantly changed. The relative density of p-Akt in control, 200 μmol/l H_2_O_2_, 200 μmol/l H_2_O_2_+ 50 m/l wogonin and 200 μmol/l H_2_O_2_+ 100 m/l wogonin were 100% ± 1.1%, 252% ± 2.1%, 112.3% ± 6.2% and 98% ± 4.8%.

## Discussion

In the present work, we evaluated the capacity of wogonin in preventing H_2_O_2_-induced oxidative stress in human APRE cell lines. Our study shows for the first time that wogonin pre-treatment can protect RPE cells from oxidative stress induced by H_2_O_2_. The use of wogonin could increase the expression of p-Akt. It could be inferred that the protective effect might be conduced via PI3K/Akt pathway.

We first evaluated the effect of wogonin on the viability of H_2_O_2_ processed ARPE-19 cells. The concentration of 100 mg/l would produce a significant effect while the concentration of 50 mg/l showed no significant change. Then we detect the anti-apoptotic effects of wogonin using Annexin V-PI double staining assays. Our results indicated that wogonin caused dose-dependent effect and the apoptotic rate in response to wogonin occurred in parallel, with Annexin V-positive cells gradually becoming Annexin-V negative. Through the western-blot, we could find that a significant p-Akt up-regulation might explain the anti-apoptotic effects of wogonin.

Wogonin is a naturally occurring mono flavonoid extracted from *Scutellaria baicalensis* radix. It has been reported that it had certain anti-oxidative effects. Chao J et al. showed that wogonin would inhibited the oxidative neuronal damage induced by H_2_O_2_ and a glutathione depleting agent D,L-buthionine [S,R]-sulfoximine. Furthermore, wogonin dramatically inhibited lipid per-oxidation initiated by Fe^2+^ and L-ascorbic acid in rat brain homogenates. It also exhibited 1,1-diphenyl-2-picrylhydrazyl radical scavenging activity. Taken together, these results demonstrated that wogonin exhibits neuroprotective actions in cultured cortical cells by inhibiting excitotoxicity and various types of oxidative stress-induced damage. The antioxidant actions with radical scavenging activity may contribute, at least in part, to the neuro-protective effects [18]. Besides, a study was conducted to investigate the possible mechanism through which wogonin inhibited the mite antigen-induced chemokine expression in human keratinocytes, HaCaT cells. It was found that wogonin could significantly suppress the mite antigen-induced TARC expression via the induction of HO1. Wogonin could induce HO1 expression, which in turn would suppresses TARC expression induced by mite antigen in human HaCaT cells [19]. However, the potential role of wogonin in the AMD is still unclear. This study provided some knowledge on this issue.

Recent evidence has revealed that PI3K/Akt pathway plays a role in the oxidative stress in the development of AMD. The regulatory role of the PI3K/Akt signal cascade in oxidative stress process appears to be cell- and ligand-specific. In a previous study, cultured human RPE cells were pretreated with medium alone, inhibitor of PI3K, Akt, or Akt/protein kinase B signaling inhibitor (API)-2, a specific Akt inhibitor, and then they were treated with H_2_O_2_ at different doses for various times to determine whether a oxidant model influences Akt activation and whether Akt activation promotes RPE cell survival. The result demonstrated that a model oxidant, H_2_O_2_, induces PI3K expression and thereby activates Akt vitality. Akt activation enhances RPE cell survival and thus may protect RPE cells from oxidant-induced cell death under normal circumstances and in abnormal states such as AMD [20]. Byeon SH et al. showed that hydrogen peroxide-induced cell death was promoted by pre-treatment with VEGF-A and anti-VEGF-R2-neutralizing antibodies, but not with anti-VEGF-R1-neutralizing antibody. Phosphorylation of VEGF-R2 in RPE cells was induced by hydrogen peroxide, and pretreatment with anti-VEGF-A-neutralizing antibody inhibited phosphorylation. Phosphorylation of Akt under oxidative stress was abrogated by pretreatment with neutralizing antibodies against VEGF-A. In conclusion, autocrine VEGF-A enhanced RPE cell survival under oxidative stress in which VEGF-A/VEGF-R2/PI3K/Akt pathway is involved. Neutralization of VEGF-A signaling, as in eyes with age-related macular degeneration, may influence RPE cell survival [21]. Besides, Liu et al. provide evidence that IFN induced VEGF expression in human retinal pigment epithelial cells. Their work emphasizes that the activation of the PI-3 K/mTOR/translational pathway is important for IFN-mediated VEGF expression in RPE cells. By elucidating molecular signaling involved in this process, our findings provide further mechanistic insight into the successful clinical application of rapamycin therapy for choroidal neovascularization in AMD [22]. A recent interesting study showed that astaxanthin (AST) clearly reduced H_2_O_2_-induced cell viability loss, cell apoptosis, and intracellular generation of ROS. Furthermore, treatment with AST activated the Nrf2-ARE pathway by inducing Nrf2 nuclear localization. Consequently, Phase II enzymes NQO1, HO-1, GCLM, and GCLC mRNA and proteins were increased. AST inhibited expression of H_2_O_2_-induced cleaved caspase-3 protein. Activation of the PI3K/Akt pathway was involved in the protective effect of AST on the ARPE-19 cells.

## Conclusions

To sum up, we firstly demonstrated that wogonin reduce the apoptosis induced by H_2_O_2_. The results extended our understandings on the molecular mechanisms of wogonin inhibiting oxidative stress in AMD. Wogonin may be a potent PI3K/Akt inhibitor and may be developed as a chemotherapeutic agent for AMD therapeutics in the future.

## References

[1] Schmidt-Erfurth U, Kaiser PK, Korobelnik JF, Brown DM, Chong V, Nguyen QD, Ho AC, Ogura Y, Simader C, Jaffe GJ, Slakter JS, Yancopoulos GD, Stahl N, Vitti R, Berliner AJ, Soo Y, Anderesi M, Sowade O, Zeitz O, Norenberg C, Sandbrink R, Heier JS (2014). Intravitreal aflibercept injection for neovascular age-related macular degeneration: ninety-six-week results of the VIEW studies. Ophthalmology.

[2] Liu L, Zou J, Jia L, Yang JG, Chen SR (2014). Spectral- and time-domain optical coherence tomography measurements of macular thickness in young myopic eyes. Diagn Pathol.

[3] Yu H, Zou X, Peng L, Wang Y, Zhang C, Chen B, Zou Y (2013). Effect of soluble inducible costimulator level and its polymorphisms on age-related macular degeneration. DNA Cell Biol.

[4] Liu L, Zou J, Huang H, Yang JG, Chen SR (2012). The influence of corneal astigmatism on retinal nerve fiber layer thickness and optic nerve head parameter measurements by spectral-domain optical coherence tomography. Diagn Pathol.

[5] Chan A, Lakshminrusimha S, Heffner R, Gonzalez-Fernandez F (2007). Histogenesis of retinal dysplasia in trisomy 13. Diagn Pathol.

[6] Cruz-Guilloty F, Saeed AM, Duffort S, Cano M, Ebrahimi KB, Ballmick A, Tan Y, Wang H, Laird JM, Salomon RG, Handa JT, Perez VL (2014). T cells and macrophages responding to oxidative damage cooperate in pathogenesis of a mouse model of age-related macular degeneration. PLoS One.

[7] Ramakrishna V, Jailkhani R (2007). Evaluation of oxidative stress in Insulin Dependent Diabetes Mellitus (IDDM) patients. Diagn Pathol.

[8] Oliveira-Costa JP, Zanetti JS, Silveira GG, Soave DF, Oliveira LR, Zorgetto VA, Soares FA, Zucoloto S, Ribeiro-Silva A (2011). Differential expression of HIF-1alpha in CD44 + CD24-/low breast ductal carcinomas. Diagn Pathol.

[9] Radu RA, Hu J, Yuan Q, Welch DL, Makshanoff J, Lloyd M, McMullen S, Travis GH, Bok D (2011). Complement system dysregulation and inflammation in the retinal pigment epithelium of a mouse model for Stargardt macular degeneration. J Biol Chem.

[10] Ersoy L, Ristau T, Hahn M, Karlstetter M, Langmann T, Droge K, Caramoy A, den Hollander AI, Fauser S (2014). Genetic and environmental risk factors for age-related macular degeneration in persons 90 years and older. Invest Ophthalmol Vis Sci.

[11] Rosen RB, Hu DN, Chen M, McCormick SA, Walsh J, Roberts JE (2012). Effects of melatonin and its receptor antagonist on retinal pigment epithelial cells against hydrogen peroxide damage. Mol Vis.

[12] Schwartz DM, Fingler J, Kim DY, Zawadzki RJ, Morse LS, Park SS, Fraser SE, Werner JS (2014). Phase-variance optical coherence tomography: a technique for noninvasive angiography. Ophthalmology.

[13] Barker FM, Snodderly DM, Johnson EJ, Schalch W, Koepcke W, Gerss J, Neuringer M (2011). Nutritional manipulation of primate retinas, V: effects of lutein, zeaxanthin, and n-3 fatty acids on retinal sensitivity to blue-light-induced damage. Invest Ophthalmol Vis Sci.

[14] Qi FH, Wang ZX, Cai PP, Zhao L, Gao JJ, Kokudo N, Li AY, Han JQ, Tang W (2013). Traditional Chinese medicine and related active compounds: A review of their role on hepatitis B virus infection. Drug Discov Ther.

[15] Su GY, Yang JY, Wang F, Ma J, Zhang K, Dong YX, Song SJ, Lu XM, Wu CF (2014). Antidepressant-like effects of Xiaochaihutang in a rat model of chronic unpredictable mild stress. J Ethnopharmacol.

[16] Lin MG, Liu LP, Li CY, Zhang M, Chen Y, Qin J, Gu YY, Li Z, Wu XL, Mo SL (2013). Scutellaria extract decreases the proportion of side population cells in a myeloma cell line by down-regulating the expression of ABCG2 protein. Asian Pac J Cancer Prev.

[17] Li Z, Dong X, Liu H, Chen X, Shi H, Fan Y, Hou D, Zhang X (2013). Astaxanthin protects ARPE-19 cells from oxidative stress via upregulation of Nrf2-regulated phase II enzymes through activation of PI3K/Akt. Mol Vis.

[18] Cho J, Lee HK (2004). Wogonin inhibits excitotoxic and oxidative neuronal damage in primary cultured rat cortical cells. Eur J Pharmacol.

[19] Lee BS, Shim SM, Heo J, Pae HO, Seo BY, Han SY, Sohn DH, Jang SI, Chung HT (2007). Wogonin suppresses TARC expression induced by mite antigen via heme oxygenase 1 in human keratinocytes. Suppressive effect of wogonin on mite antigen-induced TARC expression. J Dermatol Sci.

[20] Yang P, Peairs JJ, Tano R, Jaffe GJ (2006). Oxidant-mediated Akt activation in human RPE cells. Invest Ophthalmol Vis Sci.

[21] Byeon SH, Lee SC, Choi SH, Lee HK, Lee JH, Chu YK, Kwon OW (2010). Vascular endothelial growth factor as an autocrine survival factor for retinal pigment epithelial cells under oxidative stress via the VEGF-R2/PI3K/Akt. Invest Ophthalmol Vis Sci.

[22] Liu B, Faia L, Hu M, Nussenblatt RB (2010). Pro-angiogenic effect of IFNgamma is dependent on the PI3K/mTOR/translational pathway in human retinal pigmented epithelial cells. Mol Vis.

